# Clinical characteristics and outcomes of community acquired-acute kidney injury

**DOI:** 10.1007/s11255-023-03533-0

**Published:** 2023-03-09

**Authors:** Maggie Tso, Kamal Sud, Connie Van, Wubshet Tesfaye, Ronald L. Castelino

**Affiliations:** 1 Faculty of Medicine and Health, The University of Sydney School of Pharmacy, A15, Science Road, Camperdown, Sydney, NSW 2006 Australia; 2 Nepean Clinical School, Faculty of Medicine and Health, The University of Sydney, Sydney, NSW Australia; 3 Renal Medicine, Nepean Hospital, Sydney, NSW Australia; 4 Department of Pharmacy, Blacktown Pharmacy, Sydney, NSW Australia

**Keywords:** Acute kidney injury, Community acquired, Nephrology, Outcome assessment health care

## Abstract

**Purpose:**

Published works have reported the impact of a nephrologist intervention on outcomes for patients with hospital-acquired acute kidney injury (HA-AKI), however little is known about the clinical characteristics of patients with community-acquired acute kidney injury (CA-AKI) and the impact of nephrology interventions on outcomes in these patients.

**Methods:**

A retrospective study on all adult patients admitted to a large tertiary care hospital in 2019 who were identified to have CA-AKI were followed from hospital admission to discharge. Clinical characteristics and outcomes of these patients were analysed by receipt of nephrology consultation. Statistical analysis included descriptive, simple Chi-squared/Fischer Exact test, independent samples t-test/Mann–Whitney U test and logistic regression.

**Results:**

182 patients fulfilled the study inclusion criteria. Mean age was 75 ± 14 years, 41% were female, 64% had stage 1 AKI on admission, 35% received nephrology input and 52% had achieved recovery of kidney function by discharge. Higher admission and discharge serum creatinine (SCr) (290.5 vs 159 and 173 vs 109 µmol/L respectively, p =  < 0.001), and younger age (68 vs 79, p =  < 0.001) were associated with nephrology consultations, whilst length of hospitalisation, mortality and rehospitalisation rates were not significantly different between the two groups. At least 65% were recorded to be on at least one nephrotoxic medication.

**Conclusion:**

Our findings provide a snapshot of current practice where close to two-thirds of hospitalised patients with CA-AKI had a mild form of AKI that was associated with good clinical outcomes. While higher SCr on admission and younger age were predictors of receiving a nephrology consultation, nephrology consultations did not have any impact on outcomes.

## Introduction

Acute kidney injury (AKI) is an increasingly common problem, which is associated with high morbidity, mortality and healthcare expenditure [1, 2]. It is estimated that approximately 13.3 million people globally experience an AKI every year, leading to about 1.7 million deaths annually [3, 4]. AKI is recognised as an important risk factor for the development of chronic kidney disease (CKD), accelerated progression to kidney failure, poor health-related quality of life, and disability [4]. In Australia, in 2012–13, there were 131,780 hospitalisations, with AKI documented as the principal and/or additional diagnosis [1]. The average length of hospital stay associated with AKI was 11.4 days, as opposed to 5.6 days in people without AKI [1]. AKI was also responsible for or associated with 3.5% of all deaths in Australia in 2012 [1].

In the community setting, AKI is reported to have an incidence of 384.1 per 100,000 persons per year, [5] and when patients come to hospitals, community-acquired AKI (CA-AKI) may not be appropriately identified and treated in close to 50% of emergency presentations [6]. The International Society of Nephrology has put forward the “0by25” campaign to reduce the number of preventable deaths from AKI to zero by 2025 [4]. This initiative highlights the importance of AKI as a preventable cause of morbidity and mortality in the community. This could be achieved, for example, by targeting the use of medications that are either nephrotoxic or worsen kidney function in patients who are unwell in the community from other causes. Therefore, understanding the clinical characteristics and recognising risk factors that precipitate AKI in the community setting can provide an opportunity for early interventions to prevent AKI, especially in the at-risk populations.

Previous international and national studies have compared the epidemiology and outcomes of people with CA-AKI and hospital-acquired AKI (HA-AKI) [7–10]. These studies observed a higher incidence for CA-AKI than HA-AKI with CA-AKI patients initially having more severe AKI, yet shorter hospitalisation and better long-term survival outcomes compared to patients with HA-AKI. In both community and hospital settings, patients with AKI are not commonly seen by nephrologists and by the time they are evaluated by a nephrologist, their AKI may have progressed to a more severe form [3, 11]. Although some studies have assessed the impact of a nephrologist intervention on outcomes of patients with HA-AKI [12–15], little is known on the same in patients with CA-AKI.

Hence, the aim of this study was to determine clinical characteristics and outcomes of patients hospitalised with CA-AKI and the impact of nephrology consultations on clinical outcomes, such as recovery from AKI, length of hospitalisation and in-hospital mortality.

## Methods

### Study design and participants

This retrospective audit targeted adult patients admitted to a large tertiary care hospital in New South Wales, Australia, between 1st January and 31st December 2019 who had two or more serum creatine (SCr) measurements during their hospital admission. We used the diagnosis code as per the SNOMED clinical terms, an internationally recognised clinical terminology system used by New South Wales Health for coding electronic medical records [16]. The following terms were used for the initial inclusion of patients: ‘acute kidney injury’, ‘acute renal impairment’, ‘end stage kidney disease’, ‘ESCRF – end stage chronic renal failure’, ‘chronic kidney disease’, ‘acute on chronic renal failure’, ‘impaired renal function’, ‘CKD – chronic kidney disease’ and ‘kidney disease’. Patients were then classified as having CA-AKI if the admitting team’s clinical evaluation mentioned possibility of AKI in the admission notes and/or discharge summary. Patients were excluded if they: were on dialysis, were not suspected of having an AKI diagnosis on admission, had a planned elective surgery admission, had less than two available SCr values during hospitalisation, had developed AKI during hospitalisation, did not fulfill Duff and Murray’s [17] proposed retrospective diagnosis and staging of AKI, or were under palliative care. This study was approved by the institutional local health district human ethics committee (Approval number: 1902–09).

### Data description and collection

We extracted patients’ demographic characteristics, comorbidities, and relevant clinical information from the electronic medical records. Comorbidity burden was categorised based on the Charlson Comorbidity Index (CCI) [18], while medications were categorised as per the Anatomical Therapeutic Chemical (ATC) 2021 classification system [19]. The use of nephrotoxic medications [20, 21] and those that may worsen kidney function especially in patients who are acutely unwell were recorded. The latter group of medications are often termed as SADMANS drugs (S = sulfonylureas, A = angiotensin-converting enzyme inhibitors, D = diuretics, M = metformin, A = angiotensin receptor blockers, N = non-steroidal anti-inflammatory, S = sodium–glucose co-transporter-2 inhibitors) [22]. SCr values at admission, discharge and, where applicable, from the past 12 months were recorded to enable determination of AKI and assessment of recovery from it. We also recorded information on length of hospitalisation, intensive care unit (ICU) stay, time to nephrology consultations post admission (early < 48 h or late > 48 h), and in-hospital mortality, if any. Any documentation on the need for dialysis either during hospitalisation or continuing at discharge were recorded. Number of subsequent hospital readmissions within 6 months and between 6–12 months post-index hospital admission with CA-AKI were also collected.

### Definitions

The Kidney Disease Improving Global Outcomes (KDIGO)[23] criteria was used to define and categorise the stages and severity of AKI for those patients with a baseline SCr measured within the past 12 months of admission. Duff and Murray’s conceptual model of proposed retrospective diagnosis and staging of AKI criteria was used for patients without a baseline SCr measured within 12 months prior to admission [17]. AKI was categorised by comparing the minimum SCr value within 7 days of admission with that of the reference (first/admission) value. Recovery from AKI was based on at least a 33% reduction in SCr from admission value within 48 h (early recovery) or 48 h to 7 days (delayed recovery) [17]. We classified patients who had a < 32% reduction at 7 days as partial recovery with progression to acute kidney disease (non-recovery of AKI that persisted from 7 to 90 days) with or without CKD [17]. For the purpose of this study nephrology consultations included all patients admitted under the care of the renal team or when nephrology were consulted.

### Outcomes

We primarily assessed the following major outcomes: recovery rates from AKI (both early and delayed) and rates of and time to hospital readmission within 6 months and between 6 and 12 months of the index hospital admission. The first hospital admission during the study period (January-December 2019) was defined as the index hospital admission and used to determine time to hospital readmission. Additional outcomes, such as length of hospitalisation, rates of ICU transfer and length of stay in the ICU, dialysis during hospitalisation, and in-hospital mortality, were recorded and stratified by receipt of nephrology consultation. We also explored factors associated with receiving a nephrology consultation.

### Statistical analysis

Statistical analysis was performed using SPSS Statistics for Windows, Version 25.0 (Armonk, NY: IBM Corp.). Descriptive statistics of important demographic and clinical characteristics of patients were presented in tabular format. Mean and standard deviation (SD) or median and interquartile range (IQR) were reported, depending on normality of data distribution for each variable. Patient demographic and clinical variables were compared between patients who had nephrology consultation during their hospital stay versus those who did not. For categorical variables, a simple Chi-squared or Fischer Exact test was applied depending on the number of observations for each cell. Continuous variables were compared between the two groups using the independent samples t-test or Mann–Whitney U test depending on normality of data distribution. Proportion of patients who experienced different clinical outcomes were presented and these were compared between patients with and without nephrology consultation using the Chi-squared test for categorical variables and Mann–Whitney test for continuous variables.

To identify factors associated with receiving nephrology consultation, we performed a binary logistic regression categorising patients into ‘yes’ (for those with at least one consultation) and ‘no’ categories. For the logistic regression, we included relevant patient and clinical factors that showed significant association in the descriptive analysis and those that were deemed clinically relevant. Models that included variables such as patient age, comorbidity, duration of hospitalisation, admission SCr and presence of pre-existing CKD and dementia were performed independently to identify factors associated with receipt of nephrology consultation. To avoid the compounding effect of pre-existing CKD on admission SCr values, we performed two regression models. Model 1 included both pre-existing CKD and admission SCr values in the regression analysis, while Model 2 included only pre-existing CKD. For all analyses, statistical significance was set at *P* value of < 0.05.

## Results

**Fig. 1:**
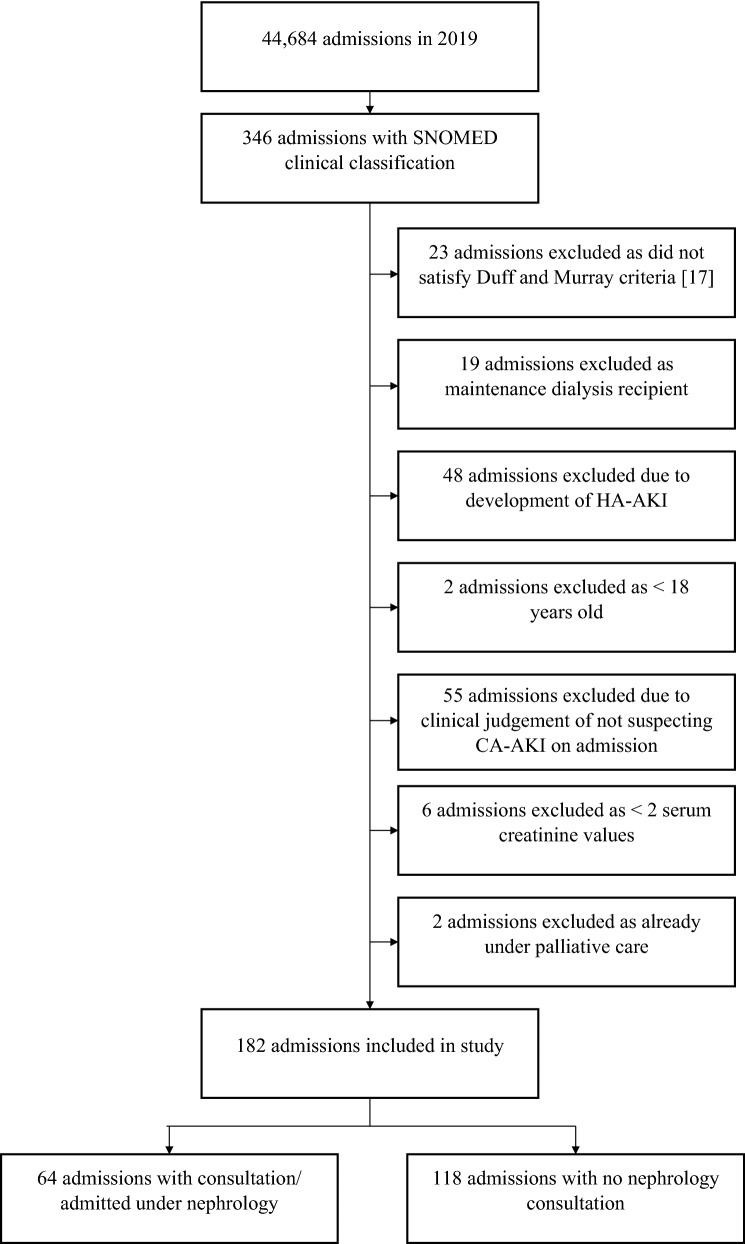
Flow diagram of admission episodes analysed in the 12-month study period

Of the 346 patients identified using the clinical coding for the targeted study period, 182 fulfilled the study inclusion criteria (see Fig. 1).

### Baseline clinical characteristics

The baseline demographic and clinical characteristics of the cohort are presented in Table 1. Mean age (SD) was 75 (14) years and 41% (n = 75) of patients were female. The median (IQR) number of medications taken by patients was 7 (4–11) and the top 5 most common medications these patients used based on ATC subgroup included: A02 drugs used in diabetes (n = 134; 73.6%), A10 antithrombotic agents (n = 179; 98.4%), B01 drugs for acid related disorders (n = 139; 76.4%), C10 lipid modifying agents (n = 119; 65.4%), C03 diuretics (n = 90; 49.5%). Amongst these there were 119 patients (65%) recorded to be on at least one nephrotoxic medication and 135 patients (74%) on at least one medication from the SADMANS group of drugs. The median (IQR) CCI score was 3 (2–5), with hypertension (n = 134; 74%), diabetes (n = 106; 58%) and pre-existing CKD (n = 73; 40%) identified as the most common comorbidities.

**Table 1:** Baseline characteristics of study population at the time of AKI (n = 182)

Characteristics*	All patients (n = 182)	Nephrology consultation^†^ (n = 64)	No nephrology consultation (n = 118)	*P*
Mean age (yr) ± SD	75 (**± **14)	68 (**± **14)	79 (**± **12)	< 0.001
Age > 75 yr (n %)	106 (58)	22 (34)	84 (71)	< 0.001
Gender; Male (n %)	107 (59)	41 (64)	66 (56)	0.29
Admission SCr (µmol/L)	182.5 (141–281.5)	290.5 (189–463)	159 (126–215)	< 0.001
Discharge SCr (µmol/L)	121 (90–174)	173 (110–283.5)	109 (79.5–139)	< 0.001
Number of medications	7 (4–11)	7.5 (4–10)	7 (4–11)	0.78
Charlson Comorbidity Index (CCI)	3 (2–5)	3 (1.25–4)	3 (2–5)	0.37
Comorbidities - (n %)				
Congestive heart failure	33 (18)	10 (16)	23 (19)	0.52
Peripheral vascular disease	23 (13)	6 (9)	17 (14)	0.36
Cerebrovascular disease	34 (19)	13 (20)	21 (18)	0.68
Lung disease	33 (18)	6 (9)	27 (23)	0.03
Cancer	40 (22)	11 (17)	29 (25)	0.25
Dementia	26 (14)	3 (5)	23 (19)	0.01
Diabetes	106 (58)	39 (61)	67 (57)	0.59
Hypertension	134 (74)	45 (70)	89 (75)	0.45
Pre-existing CKD	73 (40)	31 (48)	42 (36)	0.09
Renal Artery Stenosis	5 (3)	1 (0.02)	4 (0.03)	0.66
Stage of AKI on admission (n %)				
Stage 1	117 (64)	30 (47)	87 (74)	< 0.001
Stage 2	34 (19)	16 (25)	18 (15)	
Stage 3	23 (13)	16 (25)	7 (6)	
Unknown/indeterminable	8 (4)	2 (3)	6 (5)	
Nephrotoxic medications [20,21],	119 (65)	47 (73)	72 (61)	0.09
Use of SADMANS drug [22]	135 (74)	52 (81)	83 (70)	0.11

^†^Nephrology consultation also includes patients admitted under renal medicine speciality

*Results are presented as median (IQR) unless otherwise described

On admission, nearly two-thirds of the patients (n = 117; 64%) were classified as having stage 1 AKI, slightly less than a third (n = 57; 32%) of patients were classified as having stage 2 or 3 AKI and the stage of AKI for 4% (n = 8) were unknown. These eight patients had a baseline SCr within 12 months prior to admission, however, the baseline SCr was not ≥ 50% or ≥ 26.5 µmol/L higher than admission SCr value per KDIGO criteria or was not at least 0.66 times different to admission SCr as proposed by Duff and Murray for retrospective diagnosis. Therefore, staging of AKI on admission for these patients was not determined.

The baseline clinical characteristics of patients with CA-AKI stratified by nephrology consultation is presented in Table 2. Over one third, 64 (35%) patients, had received a nephrology consultation during their hospital admission. Excluding those admitted under the Nephrology team, a total of 39 (21%) patients had a nephrologist consultation, and 26 (67%) of these had received consultation within 48 h of hospital admission. Patients with higher admission or discharge SCr were more likely to have a nephrology consult and/or were admitted under the Nephrology team. Both admission (290.5 µmol/L [189–463] vs 159 µmol/L [126–215]) and discharge SCr (173 µmol/L [110–283.5]) vs 109 µmol/L [79.5–139]) values were significantly higher in patients who received a nephrology consultation than those who did not.

**Table 2:** Clinical outcomes of patients with community-acquired AKI by nephrology consultation

Outcome variable*	All patients (n = 182)	Nephrology consultation (n = 64)	No nephrology consultation (n = 118)	*P*
Recovery (n %)	94 (52)	38 (59)	56 (47)	0.12
Early (within 48 h)	49 (27)	15 (23)	34 (29)	0.04
Delayed (48 h-7 days)	45 (25)	23 (36)	22 (19)	
Duration of hospitalisation (days)	7 (4–12)	8 (5–15)	6 (3–12)	0.09
ICU transfer (n %)	22 (12)	11 (17)	11 (9)	0.12
ICU stay duration (days)	4 (2–7.5)	4 (3–11)	2 (1–7)	0.13
Mortality (n %)	12 (7)	4 (6)	8 (7)	0.58
Hospital readmission				
Within 6 months (n %)	106 (58)	39 (61)	67 (57)	0.47
Within 6–12 months (n %)	21 (12)	6 (9)	15 (13)	
No readmission within 12 months	55 (30)	23 (36)	32 (27)	
Discharge SCr (µmol/L)	121 (90–174)	173 (110–283.5)	109 (79.5–139)	< 0.001
Received dialysis	8 (4)	7 (11)	1 (1)	< 0.05

*Results are in median (IQR) unless described otherwise

### Outcomes

About half of included patients (n = 94; 52%) had achieved recovery of kidney function – of these, 49 (52%) achieved early reversal within 48 h of admission and 45 (48%) achieved delayed reversal between 48 h and 7 days of admission. Eighty-eight (48%) patients had a partial recovery. Fifty-nine percent of patients who received nephrology consultation recovered versus 47% of patients who did not have a nephrology consultation, however this difference was not statistically significant (p = 0.12). A higher rate of delayed recovery was observed in those with nephrology consultation than those without, 36% (n = 23) vs 19% (n = 22) (p = 0.04). Interestingly, amongst patients without nephrology consultation, early recovery (n = 34, 29%) was observed more than delayed recovery (n = 22, 19%) (p = 0.04).

Other outcomes such as length of hospitalisation, transfer to ICU during admission, duration of ICU stay and mortality between the two groups were comparable and not statistically significant. Of the original cohort, 127 (70%) patients were re-admitted within 12 months of discharge; 106 (58%) patients were re-admitted to the hospital within 6 months, of which 39 (61%) had received nephrology consultation during their index admission due to CA-AKI, and 21 (12%) patients were re-admitted within 6–12 months, of which 6 (9%) had received nephrology consultation or were admitted under the nephrology team during their index admission. Rehospitalisation rates did not differ between those who had input from nephrologists and those who did not. Of the 8 (4%) patients that received dialysis during their hospital stay, four received dialysis temporarily during their ICU admission while the other four required ongoing dialysis after discharge.

Predictors of receiving nephrology consultation are presented in Table 3. While pre-existing CKD was associated with nephrology consultations on univariate analysis, only age (OR 0.94; 95% CI 0.91–0.97) and admission SCr (OR 1.01; 95% CI 1.00–1.01) were significantly associated with receiving nephrology input on multivariate analysis in Model 1. When admission SCr was excluded from the regression analysis (Model 2), pre-existing CKD was a significant predictor of receiving nephrology consultation(s) on multivariate analysis.

**Table 3:** Predictors of receiving nephrology consultation

Variable	Unadjusted estimate (95% CI)	P-value	Adjusted estimate (95% CI)	P-value
Age	0.93 (0.91–0.96)	< 0.001	0.94 (0.91–0.97)	< 0.001
Admission SCr	1.01 (1.00–1.01)	< 0.001	1.01 (1.00–1.01)	< 0.001
CCI	0.94 (0.84–1.07)	0.36	0.99 (0.82–1.19)	0.89
LoS	1.01 (0.98–1.03)	0.63	1.00 (0.96–1.04)	0.97
Pre-existing CKD	1.7 (0.92–3.15)	0.09	1.73 (0.65–4.62)	0.27
Pre-existing CKD^¥^	1.7 (0.92–3.15)	0.09	3.27 (1.39–7.68)	0.07
Dementia	0.2 (0.06–0.71)	0.01	0.23 (0.04–1.23)	0.09

*CCI* Charlson Comorbidity Index; *LoS* length of hospital stay

^¥^Pre-existing CKD was considered in a model (Model 2) that did not include admission SCr

## Discussion

This study presents the clinical characteristics of patients with CA-AKI who were admitted to a tertiary Australian hospital over a 12-month period. CA-AKI requiring hospitalisations occurred in predominantly older adults with a moderate level of comorbidities including CKD and more than half (65%) of the patients were on at least one nephrotoxic/SADMANS medication. Close to two-thirds of patients had a mild AKI that was associated with good clinical outcomes. While higher SCr on admission, pre-existing CKD and younger age were predictors of receiving a nephrology consultation, nephrology consultation did not have any impact on clinical outcomes.

Characteristics of our study population are comparable to study populations described in similar previous studies that reported older patients were more likely to experience CA-AKI and that people with CA-AKI were likely to also have comorbidities including hypertension, CKD, diabetes and ischaemic heart disease [6–8, 10, 24]. Further, the moderate overall severity of comorbidities (based on average CCI scores) was comparable to that reported by two previous studies [8, 25]. A substantial proportion of patients in our study had a pre-existing CKD, which is consistent with previous studies that reported pre-existing CKD rates ranging from 21.8% to 53% [6–8, 10, 24, 26]. These findings collectively indicate that CA-AKI is more likely to occur in people with a history of CKD, reaffirming the notion that pathophysiology of AKI and CKD are interlinked and are likely to promote one another [27].

Nearly two-thirds (64%) of our study patients had mild AKI (stage 1), which is comparable to another Australian study conducted in a large metropolitan quaternary referral centre that found 68% of patients presented at hospital with stage 1 CA-AKI [7]. However, this is higher than that reported by a study in the United Kingdom which observed 42% of patients with stage 1 AKI and 58% of patients with stage 2 and 3 AKI [10]. The higher proportion of more severe forms of AKI reported in this study may explain the significantly higher mortality rate of 45% [10] compared to the 6.6% mortality rate we found. Another study conducted in Taiwan [8] had an in-hospital mortality rate of 26%, which could also be explained by the higher percentage of patients in the kidney failure stage per RIFLE criteria (risk, injury, failure, loss of function, and end stage of kidney disease). Other slightly higher in-hospital mortality rates were observed in studies from the United States (11.5%) [24] and China (9.7%) [25]. Our in-hospital morality rate is consistent with another large-scale Australian study that reported a mortality rate of 6.2% in people with CA-AKI [7].

The baseline clinical characteristics of included patients with and without nephrology consultation were comparable for all recorded characteristics except for age, admission and discharge SCr and stage of AKI on admission. In line with findings from previous studies, our descriptive analyses revealed that referral for nephrology consultation was associated with a more severe form of AKI (patients who had a nephrology consultation, on average, had higher SCr values both at hospital presentation and discharge), a delayed kidney function recovery and longer hospitalisation and ICU stay [12, 13, 28]. This has been confirmed in our regression analyses where admission SCr values or pre-existing CKD were identified as predictors of nephrology consultation. Age, on the other hand, showed an inverse association with nephrology consultation, indicating relatively younger patients were more likely to be referred for consultation. Overall, these findings collectively indicate that referral to nephrology consultation were understandably prompted by the presence of higher SCr measurements at hospital presentation. While it is understandable that people with CKD will also have higher SCr measurements, the association between admission SCr and nephrology consultation on a regression model persisted even after adjusting for pre-existing CKD, which may indicate that referral decisions are mainly made based on acute changes in SCr in these settings. The finding on the link between admission SCr and nephrologist referral is also consistent with our previous work in people with HA-AKI [29], that reported people receiving nephrology consultation were more likely to have higher SCr on admission and at hospital discharge, experience more severe forms of AKI, and had longer length of hospitalisation than those without a nephrology consultation.

Since close to two-thirds of patients in our study had mild AKI, we were unable to demonstrate that nephrology consultation led to a statistically significant difference in overall recovery rate, length of hospitalisation and in-hospital mortality. It is worth noting, however, when we look at people with delayed recovery separately, those with nephrology consultation had a higher chance of recovery than those without. This is likely because of the increased chance of referral among people who have not achieved kidney function recovery within the first few days of admission.

Our study had a total of 39 (21%) patients who had a nephrology consultation likely because of the predominantly mild cases of AKI in our cohort. Small percentages of nephrology referrals have also been observed in other studies. Stucker and colleagues reported that only 11 (3%) requests were made for a renal consultation in the emergency department [6]. Wonnacott and colleagues observed nephrology referral in 85 (8.3%) patients, consisting of 10.3% from the CA-AKI group and 4.2% from the HA-AKI group [10]. This study observed patients with CA-AKI were more likely to be referred to nephrology, however did not examine the impact of nephrology referral on clinical outcomes. Similar to our previous study on patients with HA-AKI [29], the current study showed nephrology consultation did not significantly impact on clinical outcomes including mortality, length of hospitalisation or requiring transfer into ICU.

Evidence indicates that exposure to nephrotoxic medicines is associated with severity of AKI [8], highlighting the need to monitor or temporarily withhold certain medications to reduce adverse outcomes, especially in those with pre-existing CKD or who are acutely ill. Our findings indicate that the majority of patients were on either nephrotoxic (65%) and/or medications that may worsen kidney function (SADMANS) (74%). The use of these medications observed in our study is higher than that reported previously targeting similar patient populations. Hsu et al. [8] observed a quarter of study participants, while Hu et al. [25] reported 57% of patients taking at least one nephrotoxic medication [25].

The altered pharmacokinetics and pharmacodynamics in CKD predisposes patients with pre-existing CKD to an increased risk of adverse medication events [30]. While we did not perform any causality assessment in our study between the use of medications and AKI incidents, several guidelines provide guidance around avoidance of nephrotoxic medications and medications that may worsen kidney function (SADMANS) in people with pre-existing CKD [31–36]. These guidelines, however, are largely based on expert consensus rather than empiric evidence [31–38]. Therefore, future studies should investigate the degree to which these medications contribute to CA-AKI with the goal of preventing hospitalisations and other related complications due to potential use of these medications.

While our study findings add to the current understanding of the characteristics of patients hospitalised with CA-AKI in the Australian setting [7, 29], these findings should be interpreted with caution, considering the study limitations. The retrospective nature of the study design targeting a small sample size from a single centre may restrict generalisability of the study findings to other populations or settings. While the use of unique identifiers for retrieving patient information enabled access to readmission data within the health district, it is possible we may have missed this information if patients were readmitted to a different health district or setting. The potential limitation associated with the coding of diagnostic clinical terms into electronic medical records could affect the actual number of patients with a CA-AKI diagnosis [11, 39, 40]. The causes of CA-AKI were not analysed, which may limit our ability to understand modifiable causes such as the use of certain high-risk medications. The definitions of AKI we used is based exclusively on SCr changes unaccompanied by urinary output, and hence may not capture the true extent, severity and time to recovery in these patients. Most studies determined the severity of AKI using an assumed baseline SCr [10, 24, 41–43]. The most accepted definition of baseline creatinine is the most recent outpatient SCr value 7 to 365 days before hospital admission to distinguish non-recovery from pre-existing CKD [44]. In our study, a baseline SCr was not available for 20 (11%) of patients, a limitation that can impact the accuracy of determining the true incidence of AKI. Our study observed patients’ kidney function until time of discharge and does not provide insight on long-term outcomes. Nonetheless, the findings of this study add to the existing knowledge of clinical outcomes in patients hospitalised with CA-AKI and the role of nephrologist interventions in an Australian context.

## Conclusion

Findings from this study provide a snapshot of current clinical practice and the epidemiology and characteristics of hospitalised patients with CA-AKI in an Australian setting. Two-thirds of patients with CA-AKI needing hospitalisation were on nephrotoxic medications/SADMANS group of drugs in the community, highlighting the potential area for interventions to reduce the incidence of AKI in the community. Nearly two-thirds of patients hospitalised with CA-AKI had a mild form of AKI that was associated with good outcomes. Whilst higher SCr on hospital admission and younger age were identified as predictors of receiving a nephrology consultation, nephrology consultations did not have an impact on outcomes. Future studies should focus on exploring known modifiable risk factors of AKI including the use of certain medications during acute illness using large scale cohorts. Patient education on self-management practices, especially in people at risk of developing AKI has the potential of reducing CA-AKI and associated morbidity and healthcare costs. Further evidence is also required to better understand the impact of nephrology consultations on outcomes of CA-AKI.

## Data Availability

The data used to support the findings of this study is available from the corresponding author upon request.
